# A step towards non-invasive characterization of the human frontal eye fields of individual subjects

**DOI:** 10.1186/1753-4631-4-S1-S11

**Published:** 2010-06-03

**Authors:** Andreas A Ioannides, Peter BC Fenwick, Elina Pitri, Lichan Liu

**Affiliations:** 1 Laboratory for Human Brain Dynamics, AAI Scientific Cultural Services Ltd., Nicosia, Cyprus; 2 Kings College Institute of Psychiatry, London, UK

## Abstract

**Background:**

Identifying eye movement related areas in the frontal lobe has a long history, with microstimulation in monkeys producing the most clear-cut results. For humans, however, there is still no consensus about the location and the extent of the frontal eye field (FEF). There is also no simple non-invasive method for unambiguously defining the FEF in individual subjects, a prerequisite for clinical applications. Here we explore the use of magnetoencephalography (MEG) for the non-invasive identification and characterization of FEF activity in an individual subject.

**Methods:**

We mapped human brain activity before, during and after saccades by applying tomographic analysis to MEG data. Statistical parametric maps and circular statistics produced plausible FEF loci, but no unambiguous definition for individual subjects. Here we first computed the spectral decomposition and correlation with electrooculogram (EOG) of the tomographic brain activations. For each of these two measures statistical comparisons were made between different saccades.

**Results:**

In this paper, we first review the frontal cortex activations identified in earlier animal and human studies and place the putative human FEFs in a well-defined anatomical framework. This framework is then used as reference for describing the results of new Fourier analysis of the tomographic solutions comparing active saccade tasks and their controls. The most consistent change in the dorsal frontal cortex was at the putative left FEF, for both saccades to the left and right. The asymmetric result is consistent with the 1-way callosal traffic theory. We also showed that the new correlation analysis had its most consistent change in the contralateral putative FEF. This result was obtained for EOG latencies before saccade onset with delays of a few hundreds of milliseconds (FEF activity leading the EOG) and only for visual cues signaling the execution of a saccade in a previously defined saccade direction.

**Conclusions:**

The FEF definition derived from microstimulation describes only one of the areas in the dorsal lateral frontal lobe that act together to plan, prepare and execute a saccade. The definition and characterization of these areas in an individual subject can be obtained from non-invasive MEG measurements.

## Background

Saccadic eye movements are quick, simultaneous movements of both eyes in the same direction. In the awake state saccades enable the eye to fixate sequentially on parts of the visual field. During the pre-saccadic period the eyes must be exactly coordinated to keep fixating on one place in the visual field, and then quickly the eyes are moved so that they land in synchrony on the same part of the visual field. In addition visual information from the old and new fixations must be integrated to provide the continuity of perception that characterizes primate vision. It is therefore no surprise that large areas of the cortex, mid-brain, cerebellum and brainstem are all part of the oculomotor system [1]. When saccades are cued by an auditory or visual cue they usually start a few hundreds of milliseconds after the cue onset and typically last less than 100 ms. Here we studied the saccade-related eye fields in the frontal lobes of the brain. These areas lead the hierarchy of the oculomotor system and play a critical role in planning and initiating the eye movements. They are therefore sensitive indicators of the integrity of brain function in general and the frontal lobe in particular. Psychiatric disorders, including schizophrenia and depression are associated with eye movement dysfunction, especially during sleep [2]

Early studies have indicated that eye movements can be elicited by electrical stimulation of large parts of the frontal lobe in both monkeys [3,4] and humans [5,6]. The development of microstimulation methods allowed “precise” definition of eye movement areas in the frontal cortex. One particular area, the Frontal Eye Field (FEF), is well defined anatomically, cytoarchitecturally and functionally in monkeys. The FEF is on the rostral bank of the arcuate sulcus and its stimulation with currents below 50 μA produces saccades in the contralateral direction [7-9].

The introduction of positron emission tomography (PET) in the 1980s and functional Magnetic Resonance Imaging (fMRI) in the 1990s provided new tools for studying the human brain in general and saccadic eye movements in particular. Early studies identifying the “human FEF” produced consistent locations for PET [10,11] and fMRI [12-14]. Surprisingly, this putative human FEF was identified more caudal and dorsal than would have been expected from cytoarchitectonic arguments and homology with the classic FEF established by the previous meticulous animal microstimulation experiments. New experiments with fMRI in monkeys [15,16] and electrical stimulation in the lateral frontal cortex of epileptic patients [17,18] resolved the paradox. For both monkeys and humans there are at least two areas associated with eye movements in the dorsal and lateral part of the frontal cortex [19]. One corresponds to the classic FEF, defined by its ability to elicit eye movements in the contralateral direction with low current injections. The other one is more caudal and superior, seen best with fMRI in both humans and monkeys.

 The classic FEF, from the above studies, although undoubtedly an important structure, is only one of the areas in the dorsal part of the frontal lobe making up the network for the planning, control and initiation of eye movements. The studies reviewed above also suggested that there is a fairly strict homology between the structures identified in monkeys and humans. In this work we aimed to define the human homologue of the classic FEF and possibly other eye movement areas in the immediate vicinity of the dorsal and lateral aspects of the cortex. We will not consider the eye field areas close to the midline, namely the cingulate eye field [20] and supplementary eye field [21], or the areas in the more ventral part of the lateral frontal cortex. Here we will focus on the area that is roughly in front of the dorsal part of the motor cortex, at the level of the hand motor area. This is the area where, as we described above, recent studies in monkeys and humans have placed the two main frontal eye field areas.

From the theoretical point of view, the location and characterization of the human eye fields has its own intrinsic scientific importance. Added to this a better understanding of the role of each eye field may help us understand pathological conditions that are associated with changes in eye movement properties, and so possibly lead to clinical applications. It is well known that depressive illnesses, bipolar disorder, schizophrenia [22] and dementia [23] produce changes in eye movements. What is not clear is the frontal cortical component to these alterations, and how this may change with the severity of the disease and the likely therapeutic response to treatment. Neither a general definition of eye fields in terms of average locations across subjects nor a definition based on invasive stimulation is useful for treating individual patients. This is particularly important when considering epilepsy surgery for foci in the frontal cortical area. Detailed knowledge of the exact location of the eye movement fields in that patient will prevent damage to these structures. To this end, a non-invasive definition of the various eye fields for each individual subject is necessary. In this work we have taken a first step in this direction. Our starting point is the framework made up of human anatomical landmarks as defined by recent meta-analytic approaches linking monkey and human results [17,19]. We then place in the same framework the FEF loci defined by neuroimaging studies including our earlier MEG studies of saccadic eye movements [1,24]. Finally we show new and detailed analysis of a set of high sampling MEG recordings from one subject performing saccadic tasks. We present the results in the anatomical context established by the earlier studies. The new analysis identifies highly significant activations at loci corresponding to the FEFs defined by cortical stimulation and fMRI experiments. Our results reported here offer the first non-invasive clear-cut definition of FEFs in an individual subject and provide a rich characterization of the evolution of their activity over time during different eye movement tasks.

## Methods

### Anatomical landmarks for the definition of FEF in earlier studies

The recent studies reviewed in the Introduction have identified at least two “human FEFs”. One corresponds to the classic FEF definition obtained by microstimulation in the monkey (and more recently in humans). The other is identified more prominently in fMRI studies from both humans and monkeys. We summarize these results and show them superimposed on the MRIs of one of our subjects using the key anatomical landmarks as references. The loci from the different studies are first transformed to a common Talairach space and then back transformed on to the MRI of the same subject. We are thus able to define the important sulci with the results of previous studies from our groups and others. The results of this new analysis can then be interpreted in the context of the earlier studies.

### Experiments and subjects

The details for the two main MEG experiments (EXP1 and EXP2) studying saccades are described elsewhere [1,24] and so are only briefly discussed here. In EXP1 the MEG signal was recorded for three types of saccades with a sampling rate of 625 Hz [1]. The first two saccade types were in the waking state, one following an auditory cue every 4 seconds and the other self initiated  with the same tempo. We chose the tempo of one saccade every 4 seconds because it appeared easy and “natural”. The third type was composed of saccades during rapid eye movement (REM) sleep. REM saccades were chosen from a whole-night MEG recording with properties similar to those studied in the awake state. The data from three subjects  for the three types of saccades have been analyzed and the results reported in two publications [1,25].

EXP2 was designed to disentangle aspects of saccades that could not be separated by the design of EXP1. EXP2 consisted of two separate sets of measurements. In the first set of measurements (EXP2A) four subjects were recorded with the same sampling rate of 625 Hz as in EXP1. An eye tracker and EOG were used to record the ocular muscle activity and the eye movements. In EXP2A we used a set of visual cues to specify whether a saccade was to be made or not (Move, M-cue; GO/NOGO), the direction of the saccade (Direction, D-cue; Left/Right) or the initiation of the saccade (Action, A-cue). The (M+D+A) information was provided at the same or different time, either in one step by a single cue, or in three steps separated by a few seconds from each other. There were five separate types of temporal sequences, one control condition (passive viewing of stimuli; Type0) and four saccade conditions (Type1-4). In the first two active sequences (Type1-2), the M, D and A cue were presented with a few seconds between each other: in Type1 the sequence was D → M → A and in Type2 the sequence was M → D → A. In the other two sequences (Type3-4) the information was collapsed into one cue. Type3 sequence had only GO trials so the cue provided simultaneously the (D+A) information. Type4 sequence had GO and NOGO trials so the cue provided simultaneously the full (M+D+A) information. Of special interest and relevant to the results reported here are the cues releasing or inhibiting saccades (Cue3 for Type3-4, Cue6 for Type2), and the cue providing purely directional information (Cue4 in GO trials of Type2). A detailed description and diagrams of the stimuli and temporal sequences can be found in [24].

In the second part of EXP2 (EXP2B) we recorded the MEG signal from two subjects with a higher sampling rate of 2083 Hz. To avoid lengthy experimental time and subject over-training, only a subset of sequences was used in EXP2B. In one subject, who also participated in EXP2A with simultaneous MEG, EOG and eye tracker recordings, we used sequences of Type0 (control) and Type3-4. No eye tracking was used on this occasion. For the second subject (did not participate in EXP2A), we recorded MEG signals together with EOG for sequences of Type0, 2, 3 and 4, 2 runs for each type. In the present paper we will report only results from the new analysis of the data from the second subject (hbd050) in EXP2B.

All our subjects were healthy, right handed males, with normal visual acuity, binocular vision and normal optic fundi. They had no history of neurological or psychiatric illness or drug abuse.

### Quantitative tomographic analysis of brain activity

The previous results discussed in this paper together with the results of the new analysis on a single subject rely on meta-analysis of tomographic estimates of activity extracted for each timeslice of each single trial of the MEG data. Magnetic field tomography (MFT) [26] was used throughout to compute these estimates of brain activity as described in our earlier studies [1,24]. Briefly MFT produces a tomographic estimate of the vector field for the current density vector **J**(**r**,t) in the brain from each timeslice of data. For each timeslice, t, the continuous estimates of **J**(**r**,t) are discretized and stored for further analysis at 17x17x17 grid points covering the whole brain. At the sampling rate of 2083 Hz (sampling step of 0.48 ms), and given that for each cue presentation the MFT solutions were obtained for one second before and one second after cue onset, about 4000 or 8000 tomographic maps of **J**(**r**,t) were obtained for each single trial depending on whether one (Type0, 3-4) or two (Type2) cues were selected for the analysis. Clearly the volume of MFT solutions requires post-MFT analysis at different levels, such as averaging, statistical parametric mapping (SPM), circular statistics [27,28] as previously described [1,24]. The SPM comparisons of different saccadic tasks produced well-circumscribed foci of activity. These foci guided further inspection of the single trial MFT solutions for the definition of regions of interest (ROI) and the dominant (main) direction of the current density vector. These ROIs were used to extract time series for regional activations in each single trial and hence provide time-dependent measures of signal content within areas (e.g. signal to noise ratio) or measures of linked activity between areas (e.g. mutual information) [1,24]. Detailed analysis was performed after alignment of the MFT solutions relative to either cue or saccade onset. Here we also introduced two new measures of brain activity: spectral analysis and correlations between brain activation and auxiliary EOG channel. Each of these two measures was applied independently for each grid point producing alternative tomographic representations of brain activity. We describe the two new measures next.

### Spectral analysis of single trial MFT solutions

The current density vector **J**(**r**,t) from MFT solution at each grid point produces three timeseries, one for each Cartesian component. The Fourier transform coefficients for each component are obtained from segments of duration D (from t_1_ to t_2,_ D = t_2_ - t_1_) in a frequency range from f_1_ to f_2_ at a step Δf. At each frequency a pseudo-vector can be defined from the Fourier amplitude for each Cartesian component of **J**(**r**,t). The moduli of these pseudo-vectors are then used to make grid point by point comparisons between conditions, using SPM in exactly the same way as for the time domain, but now describing the significant changes of activity within frequency ranges. In the Results section we will report the Fourier spectra extracted from two 400 ms long single trial segments aligned to saccade onset (defined by the EOG channel). The first segment was extracted just before saccades (from -410 to -10 ms). The second segment was centered at the saccade onset (from -200 to 200 ms) and thus captured the entire saccade period, which usually lasts well below 100 ms. For each of these two segments, the Fourier spectra were computed from 3 Hz to 600 Hz at a step of 1 Hz. Then, we applied SPM analysis to compare two distributions. Each distribution consisted of the frequency amplitudes from a sliding 4-Hz window (5 values from each single trial). The centre of the window covered the range from 5 to 593 Hz with a step of 2 Hz. Statistical comparisons were made between each of the two recording runs for Type3, Type4 and the control condition (Type0), separately for saccades to the left and right. Finally, grid points showing common activations in the four SPM maps with significance level p < 0.005 (after Bonferoni correction) were delineated and placed in the context of anatomical landmarks defined earlier. These common activations were identified within an 80 Hz window. The centre of this window was from 45 Hz to 545 Hz in 20 Hz steps.

### Correlations between brain activations and auxiliary EOG channel

For each single trial aligned to the cue onset, we computed the cross correlation between brain activations and the EOG for segments of duration D=200 ms. The correlation was done for each current density component at each grid point with a relative delay, τ, between the brain activity and EOG. This resulted in cross correlation vectors **C**(τ). To remove the strong dependence of **C** on periods with large EOG or J values, we normalized **C** by dividing each of its components by the Euclidean norms of **J**(**r**,t) and EOG segments over the time range of D. The normalized correlation vectors, **Ĉ**, were computed for τ from -600 ms (i.e. brain activity leading the EOG) to 200 ms (i.e. brain activity lagging the EOG) with a step of 20 ms. Then, we applied SPM analysis to compare two distributions made of moduli of normalized correlation coefficients (Ĉ = |**Ĉ**|). The pair of distributions was from different cues of the same trial (e.g. Cue6 versus Cue4 in Type2) or same cue comparing GO with NOGO trials (e.g. Cue6 in Type2; Cue3 in Type4). The comparisons were computed for delays, τ, from -580 to +160 ms in steps of 20 ms, allowing a delay jitter of 20 ms. We will report results for two EOG reference segments: one is centered at 0, corresponding to the cue image onset and hence well before any strong EOG activity associated with saccade onset. The other is centered at 300 ms post cue image onset, corresponding to the mean time of the saccade onset, and thus likely to contain at least in some of the trials strong EOG activity related to the saccade onset.

## Results

### Characterization of FEF activity from our earlier studies

From the EXP1 data, we identified consistent foci of activity on the cortex, cerebellum and brainstem, for three subjects. Specifically, some of the cortical activity was consistent with the FEF location as far as one could judge on the basis of the landmarks of the local anatomy of each subject. The activation time courses of each area were extracted from the tomographic solutions and analyzed in single trials. The analysis showed that the entire process of saccade planning, initiation and execution seemed to merge into the imposed (0.25 Hz) rhythm that dominated the activity in each area. A more detailed analysis of the data showed that slow rhythms and fast activity contributed to brain activations and the way that these activations were linked to each other and the eye movements (as these were quantified by the fast EOG activity) [1].

From the EXP2 data, for two subjects, we identified highly significant changes of activity in all the key areas, including putative FEF, cerebellum and brainstem. Thanks to the higher sampling rate than EXP1 and the use of sequences with cues providing separate pieces of information about the saccadic task, we were able to show that the brain used each piece of information as soon as it was available using both fast transient activity, lasting only a few milliseconds, and slow activity, lasting hundreds of milliseconds [24].

### Anatomical and functional definition of human FEF

**Figure 1:**
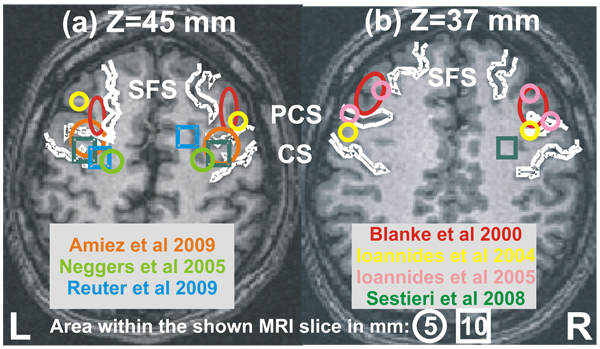
**Definition of human FEF from seven studies.** The FEF loci were transformed into the Talairach space and superimposed onto one of our subjects’ MRIs. The display is shown at two axial slices, one with Talairach coordinate of (a) Z=45 mm and one with (b) Z=37 mm. Color denotes the FEF definition from different studies (Red: Blanke et al [17]); orange: Amiez et al [19]; light green: Neggers et al [29]; green: Sestieri et al [31]; blue: Reuter et al [30]; yellow: Ioannides et al 2004 [1] and pink: Ioannides et al 2005 [24]). Shape denotes the distance between the loci and the MRI slices (circles and squares for loci within 5 mm and 10 mm of the slice, respectively). For reference, major landmarks are also shown as white outlines: central sulcus (CS), precentral sulcus (PCS) and superior frontal sulcus (SFS). The FEF definitions from each of the studies by others are represented by one set of symbols (one for left and one for right hemisphere, each marking the mean coordinates of a group of subjects used in that study), while from our studies the FEF definitions for individual subjects are shown (yellow circles in (a), yellow and pink circles in (b)).

The human FEF as defined in a series of recent studies using epicortical stimulation [17], fMRI [19,29,30], fMRI-MEG [31] and MEG [1,24] are shown on subject hbd050’s MRIs (Figure 1). In this figure, we used the anatomical landmarks as described in [19], the MNI and/or Talairach coordinates for FEF definition provided in the above five studies by others [17,19,29-31] and our two studies [1,24]. The loci defined in the Talairach space were then back-transformed to the MRI coordinates of subject hbd050 for the final display. The display is shown at two axial slices, with Talairach coordinate of (a) Z=45 mm and (b) Z=37 mm, respectively. The major sulci are represented in the same figure by heavy white outlines: the superior frontal sulcus (SFS) runs from the rostral end of each hemisphere in the anterior-posterior direction, while the pre-central (PCS) and central sulci (CS) run along a lateral to medial direction with the PCS the more anterior of the two. We use color to distinguish the FEF definitions from the seven studies. For each of the studies by others, we represent the FEF definitions by the mean location averaged across subjects for each hemisphere. For our studies, we use the FEF definitions for each of the five subjects (three subjects from EXP1 and two from EXP2, left and right hemisphere, so total of 10 FEF locations). Figure 1 shows that seven out of 10 loci either coincide or are close to the border of the definition provided by Blanke et al. [17], while one more is just behind the precentral sulcus (yellow circle in the left hemisphere in Figure 1b) and it is therefore more consistent with the FEF definition usually given by fMRI studies. The other two FEF definitions were up to 1cm more inferior to the axial slice in Figure 1b. In summary our post-MFT analysis produced FEF definitions that were broadly consistent with those derived from epicortical stimulations.

### New FEF definitions

**Figure 2:**
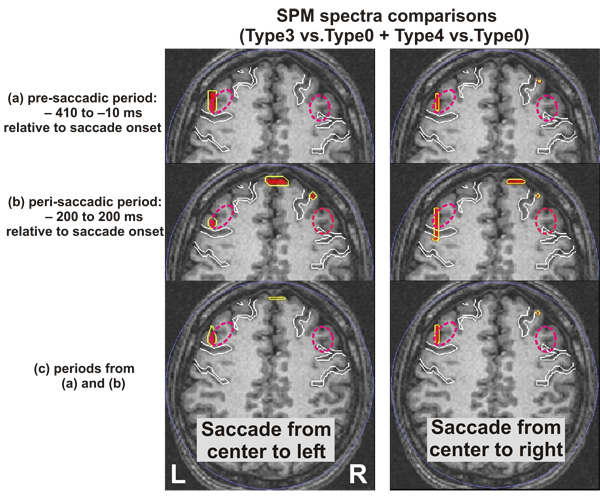
**Statistical comparison of spectra between saccades and passive viewing.** Common significant change of activity from saccades (Type3, Type4) versus passive viewing (Type0) for pre-saccadic (a), peri-saccadic (b) and both (c) periods, respectively for saccades to the left (left column) and to the right (right column). Red blob and yellow contours denote areas with p <0.005. Dotted magenta ellipsoid marks the classic FEF as defined by Blanke’s microstimuation study in humans [17] , while white outlines mark major landmarks as in Figure 1.

In our two earlier studies on eye movement, we identified activity in many other parts of the frontal cortex, not just the putative FEFs discussed above, at different times before, during and after saccades. The high variability across trials and subjects prompted us to look for quantitative descriptions of activity in the frequency domain using the procedure described in the Methods section. Statistical comparisons of the Fourier spectra between saccades and passive viewing produced clear left FEF activation for both the pre- and peri-saccadic periods. Saccade data were from Type3 and Type4, 2 runs for each Type, while passive viewing is from Type0, so there are four comparisons in total (i.e. Type3 vs. Type0; Type4 vs. Type0). Figure 2 shows the common significant change of activity from the four comparisons with a center window at 125 Hz (i.e. window range 85 to 165 Hz), for 10^0^ saccades from the center to the left (left column) and to the right (right column). The figure shows the FEF loci spreads from the classic FEF location (as defined by Blanke et al. [17]; dotted magenta ellipsoids in Figure 2) to a more posterior area for saccades to the right, especially during the saccade period (Figure 2b). These results were identified in the same area (left FEF) over a wide range of frequencies, from 85 Hz to 500 Hz for saccades to the left and from 85 to 545 Hz (the highest frequency window studied) for saccades to the right.

**Figure 3:**
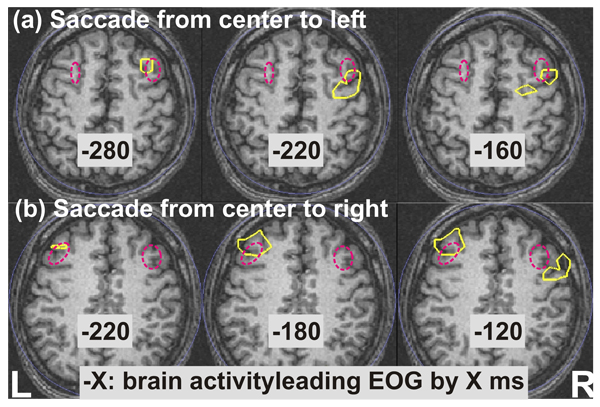
**Statistical comparison of correlations between brain activity and EOG.** The computation is made from Type2 GO trials, Cue6 (action cue) versus Cue4 (direction cue), for saccades to the left (a) and to the right (b). Results for different time delays between the brain activity and EOG are shown in different columns. Yellow contours denote the statistical significance threshold of p < 0.001. Dotted magenta ellipsoid marks the classic FEF location [17].

The definition for FEF for the single subject is best obtained from the correlation measure: contralateral FEF activity was observed clearly with delay relative to the EOG of a few hundred milliseconds (brain activity leading the EOG). As an example, we computed the correlation between brain activity and EOG for Cue4 and Cue6 from Type2 GO trials. In Type2 runs, the saccade information is given in the order of M, D and A (Move, Direction, Action cues; see Methods). Both Cue4 and Cue6 mark the onset of the same visual image but carry different saccade information to the subject. Cue4 indicates the saccade direction, while Cue6 is the action cue that subject should saccade as soon as this cue is present. We then applied the SPM analysis to the normalized correlation coefficients for Cue4 and Cue6. Figure 3 shows the SPM results (yellow contours) from the EOG reference segment centered at the cue image onset (see Methods), for saccades to the left (a) to the right (b). The figure shows clear-cut contralateral FEF loci, close to the classic FEF location (dotted magenta ellipsoids in Figure 3). Notably, in Figure 3a, as the delay between brain and EOG activity (τ) is reduced, right FEF spreads from a rostral (τ = -280 ms) to more posterior area (τ = -160 ms). This posterior area is usually defined as FEF from fMRI studies (see Figure 1a). Furthermore, similar results are observed: (1) When the EOG reference segment is centered at 300 ms post cue image onset, which corresponds roughly to the mean time of saccade onset and thus likely to contain some strong EOG activity related to the saccade onset (see Methods); (2) For comparison of Cue6 GO versus NOGO trials in Type2. Importantly, the above result is not seen for the comparison between the Cue3 GO and NOGO trials in Type4. Our results thus suggest that contralateral FEF activity is present only if the directional information is already given to the subject and the subject maintains the ocular segments in readiness for this goal while waiting for the action cue to make a saccade.

## Discussion

The overall picture that emerges from our MEG studies is a very complex one, but consistent with both the microstimulation and fMRI studies. In our previous studies, we found no simple way to define the FEFs for individual subjects: the definition cannot be guided by the latency or the laterality of the activations obtained by averaging across trials, nor from the SPM comparisons between conditions in any specific latency range in the few hundreds of milliseconds before, during or after saccade onset. Although it was possible to obtain what in retrospect appeared to be good FEF definitions (purely judging on anatomical criteria), the procedure was so cumbersome and complicated (see subsection *Complex identification of regions of interest* in the Methods section in [24]) that it cannot be routinely used in a clinical setting. There was no obvious simple sequence in the time domain that could capture adequately the variance in the single trial activations. The most ordered set of activations in our earlier studies, and also consistent across subjects, were obtained when the activity in different brain areas was linked to the EOG using mutual information (MI) analysis [1]. The MI was computed relative to EOG onset and as a function of delay between brain and EOG activations. FEF activity in the last 100 ms leads to peaks in MI corresponding to EOG activity after EOG onset. The brain activity just before saccade onset, even at the level of the brain stem gaze centers, was linked to EOG burst after saccade onset [1]. The variability across single trials was even more evident in the analysis of the second experiment. In this experiment too, the MI analysis between brain areas provided the best organizing principle for the sequence of activations [24]. In addition circular statistics analysis of fast transient responses showed that the even at the finest timescales (millisecond), we could access a rich organization that could not be adequately described by simple stacking of single trials across external markers like cue or saccade onset.

The more refined analysis reported here for a single subject begins to unravel some of the complexities associated with the role of the FEF in saccade generation. The frequency analysis shows that the changes in pre-saccadic and peri-saccadic periods compared to passive viewing of stimuli are more prominent in the left FEF for both saccades to the left and right. The one-way callosal traffic theory [32] offers one possible explanation for this asymmetry. According to this theory the decision to move the eyes begins in the major hemisphere, i.e. left hemisphere for a right-handed subject. The excess activity in the left FEF compared to the control condition could therefore be interpreted as the correlate of the decision to execute the eye movement by the left hemisphere.

The contralateral FEF is best seen in the correlation between brain activations and the EOG. The laterality of the FEF activation is not just on the contralateral side, as shown by the Fourier spectra analysis, so the purely contralateral activity is most clearly seen when the directional information is already provided and the subject simply waits for the action cue. The result is therefore clearer for comparisons between periods before a cue signaling the execution of a saccade in a given direction with periods before a cue providing information about which direction the saccade will be made (Figure 3) or when the same cue is from a NOGO trial.

Based on our results we can interpret the most widely used definition of FEF (through microstimulation experiments) in a new way. This definition fit the data because it bypasses many of the complexities that voluntary and cued saccades necessarily entail. Eye movement is the outcome of finely balanced competing actions by agonist and antagonist muscles [33]. The classic definition of FEF should then be interpreted as the identification of the cortical brain area where minimal injection of current can tip the balance in favor of a saccade.

On the other hand, the functional activations from fMRI have identified a more general region, which is likely to be involved in a more complex task than just “tipping the balance”. We know from our previous study [24] that the activity in the oculomotor system depends on linked activity at different scales, from a few milliseconds to a good fraction of a second. It is therefore impossible to study saccades in detail because of the limited time resolution of the technique. In addition to the lack of millisecond time resolution, the sluggishness of the fMRI response means that it can never isolate the activity related to saccades in just one direction. This is because after each saccade, the eye must either be held with gaze away from equilibrium or a movement must be made to return the eye to the centre. Each of these actions is likely to modify the activity in the same areas involved in the saccade generation. Our new analysis allows us to characterize the saccade-related activity within finite latency ranges using different time scales (frequencies) and isolate the part of the activity that relates to the EOG. Our new results suggest a rostro-caudal progression of the activity as the time of the saccade initiation approaches, beginning with activity in the classic FEF area as defined by microstimulation progressing later to the more posterior areas that are more prominently seen with fMRI.

We note also that saccade onset can be delayed by transcranial magnetic stimulation (TMS) [34], but it has been proved more difficult to initiate saccades using TMS. This may be a consequence of the complex spatial organization that the rather crude TMS stimulation does not fulfill, unlike microstimulation that will selectively target a discrete area. By exciting a large area TMS is likely to stimulate cells initiating and inhibiting saccades.

As in our other two studies [1,24] the lag between the FEF activity and activity in the ocular muscles, as measured by the EOG, is measured in hundreds of milliseconds and it is present already before the cue image onset and it continues through to the saccade onset (data not shown). The EOG activity before saccade onset is usually considered as random fluctuations. Our results show that this pre-saccadic EOG activity is clearly correlated with task-related FEF activity. We speculate that this pre-saccadic EOG activity reflects the preparation of ocular muscles for accurate execution of the impending saccades. The linked activity between the EOG and the brain can be captured by linear measures like correlation or non-linear ones like MI, and thus provides a sensitive tool for exploring the role of specific areas in different saccadic tasks. This speculation is supported by our other study in this volume dealing with precise measurements of each eye’s position when subjects executed saccade sequences of Type3 and Type4 [35]. The mutual information in that study was computed between timeseries for the location and velocity of each eye and thus the yoking of the two eyes was defined. Particularly relevant to our results reported here was the finding that before the initiation of saccades, the MI derived from the position information of the yoking of the two eyes was significantly higher in the Type4 (GO/NOGO trials) sessions relative to the Type3 (GO trials only) sessions.

## Conclusions

It is possible to define the human FEF(s) of individual subjects after careful analysis of the tomographic estimates of activity extracted from completely non-contact, non-invasive MEG recordings. The use of time domain information leads to the identification of either the homologue of the classic FEF identified in animals or the “other” FEF located a little more caudally and dorsally. Adding frequency analysis and especially the grid point by point correlation with the EOG can separate these two FEF subdivisions (and probably show further subdivisions). In this study we emphasized the localization and characterization of the FEF for a single subject. The generalization of the specific results we reported here cannot be assumed *a priori* for other subjects. It is likely that some of the details may change, so to reach conclusions for the population the experiments must be repeated with many subjects, taking into account the age, gender, and handedness of individual subjects.

A long term goal of our work is to provide characterization of the state of an individual and biomarkers from non-invasive measurements. Eye movements are known to be affected in many neurological and psychiatric disorders. We anticipate that clinically useful biomarkers can be defined for application to individual subjects by combining a detailed understanding of the oculomotor system anatomy and function with detailed observations of its electric and magnetic activity and even just optical recordings. The results reported here and in the companion study [35] represent the first tentative steps in this direction. For future use in clinical applications it is important to identify how reproducible these definitions are for the same individual across weeks, months and years, so that the FEF localization and functional characterization can serve as a biomarker of health and disease.

## Competing interests

The authors declare that they have no competing interests.

## Authors' contributions

AAI, LCL and PBCF designed the experiment and carried out the MEG measurements. AAI and LCL conducted the data analysis. AAI, PBCF and EP worked on the anatomical definitions of FEFs and the representation of functional definitions of FEFs from previous studies on common diagrams. AAI, LCL and EP designed and produced the figures. AAI and LCL wrote the manuscript. All authors read and approved the final manuscript.

## References

[B1] Ioannides AA Corsi-Cabrera M Fenwick PBC Portilla YD Laskaris NA Khurshudyan A Theofilou D Shibata T Uchida S Nakabayashi T MEG tomography of human cortex and brainstem activity in waking and REM sleep saccades. Cerebral Cortex 2004 14 56 72 10.1093/cercor/bhg091 14654457

[B2] Benson KL Zarcone VP Jr Rapid eye movement sleep eye movements in schizophrenia and depression. Arch Gen Psychiatry 1993 50 474 482 8498882 10.1001/archpsyc.1993.01820180076008

[B3] CROSBY EC YOSS RE HENDERSON JW The mammalian midbrain and isthmus regions. Part II. The fiber connections. D. The pattern for eye movements on the frontal eye field and the discharge of specific portions of this field to and through midbrain levels. J Comp Neurol 1952 97 357 383 10.1002/cne.900970205 12999993

[B4] Ferrier D Experiments on the basis of monkeys. Proc R Soc Lond B Bio Sci 1875 23 409 430 10.1098/rspl.1874.0058

[B5] Foerster O The cerebral cortex in man. Lancet 1931 2 309 312

[B6] Penfield W Rasmussen T The Cerebral Cortex of Man. A Clinical Study of Localization of Function. 1950 McMillan

[B7] Bruce CJ Goldberg ME Primate frontal eye fields. I. Single neurons discharging before saccades. J Neurophysiol 1985 53 603 635 3981231 10.1152/jn.1985.53.3.603

[B8] Bruce CJ Goldberg ME Bushnell MC Stanton GB Primate frontal eye fields. II. Physiological and anatomical correlates of electrically evoked eye movements. J Neurophysiol 1985 54 714 734 4045546 10.1152/jn.1985.54.3.714

[B9] Stanton GB Deng SY Goldberg ME McMullen NT Cytoarchitectural characteristic of the frontal eye fields in macaque monkeys. J Comp Neurol 1989 282 415 427 10.1002/cne.902820308 2715390

[B10] Paus T Location and function of the human frontal eye-field: a selective review. Neuropsychologia 1996 34 475 483 10.1016/0028-3932(95)00134-4 8736560

[B11] Petit L Dubois S Tzourio N Dejardin S Crivello F Michel C Etard O Denise P Roucoux A Mazoyer B PET study of the human foveal fixation system. Hum Brain Mapp 1999 8 28 43 10.1002/(SICI)1097-0193(1999)8:1<28::AID-HBM3>3.0.CO;2-T 10432180 PMC6873342

[B12] Corbetta M Akbudak E Conturo TE Snyder AZ Ollinger JM Drury HA Linenweber MR Petersen SE Raichle ME Van Essen DC A common network of functional areas for attention and eye movements. Neuron 1998 21 761 773 10.1016/S0896-6273(00)80593-0 9808463

[B13] Luna B Thulborn KR Strojwas MH McCurtain BJ Berman RA Genovese CR Sweeney JA Dorsal cortical regions subserving visually guided saccades in humans: an fMRI study. Cereb Cortex 1998 8 40 47 10.1093/cercor/8.1.40 9510384

[B14] Petit L Clark VP Ingeholm J Haxby JV Dissociation of saccade-related and pursuit-related activation in human frontal eye fields as revealed by fMRI. J Neurophysiol 1997 77 3386 3390 9212283 10.1152/jn.1997.77.6.3386

[B15] Baker JT Patel GH Corbetta M Snyder LH Distribution of activity across the monkey cerebral cortical surface, thalamus and midbrain during rapid, visually guided saccades. Cereb Cortex 2006 16 447 459 10.1093/cercor/bhi124 15958778

[B16] Koyama M Hasegawa I Osada T Adachi Y Nakahara K Miyashita Y Functional magnetic resonance imaging of macaque monkeys performing visually guided saccade tasks: comparison of cortical eye fields with humans. Neuron 2004 41 795 807 10.1016/S0896-6273(04)00047-9 15003178

[B17] Blanke O Spinelli L Thut G Michel CM Perrig S Landis T Seeck M Location of the human frontal eye field as defined by electrical cortical stimulation: anatomical, functional and electrophysiological characteristics. Neuroreport 2000 11 1907 1913 10.1097/00001756-200006260-00021 10884042

[B18] Yamamoto J Ikeda A Satow T Matsuhashi M Baba K Yamane F Miyamoto S Mihara T Hori T Taki W Human eye fields in the frontal lobe as studied by epicortical recording of movement-related cortical potentials. Brain 2004 127 873 887 10.1093/brain/awh110 14960503

[B19] Amiez C Petrides M Anatomical organization of the eye fields in the human and non-human primate frontal cortex. Prog Neurobiol 2009 89 220 230 10.1016/j.pneurobio.2009.07.010 19665515

[B20] Dum RP Strick PL The origin of corticospinal projections from the premotor areas in the frontal lobe. J Neurosci 1991 11 667 689 1705965 10.1523/JNEUROSCI.11-03-00667.1991 PMC6575356

[B21] Brinkman C Porter R Supplementary motor area in the monkey: activity of neurons during performance of a learned motor task. J Neurophysiol 1979 42 681 709 107282 10.1152/jn.1979.42.3.681

[B22] Harris MS Reilly JL Thase ME Keshavan MS Sweeney JA Response suppression deficits in treatment-naive first-episode patients with schizophrenia, psychotic bipolar disorder and psychotic major depression. Psychiatry Res 2009 170 150 156 10.1016/j.psychres.2008.10.031 19906441 PMC2792232

[B23] Crutcher MD Calhoun-Haney R Manzanares CM Lah JJ Levey AI Zola SM Eye tracking during a visual paired comparison task as a predictor of early dementia. Am J Alzheimers Dis Other Demen 2009 24 258 266 10.1177/1533317509332093 19246573 PMC2701976

[B24] Ioannides AA Fenwick PB Liu L Widely distributed magnetoencephalography spikes related to the planning and execution of human saccades. J Neurosci 2005 25 7950 7967 10.1523/JNEUROSCI.1091-05.2005 16135752 PMC6725466

[B25] Ioannides AA Kostopoulos GK Liu L Fenwick PB MEG identifies dorsal medial brain activations during sleep. Neuroimage 2009 44 455 468 10.1016/j.neuroimage.2008.09.030 18950718

[B26] Ioannides AA Bolton JPR Clarke CJS Continuous probabilistic solutions to the biomagnetic inverse problem. Inverse Problems 1990 6 523 542 10.1088/0266-5611/6/4/005

[B27] Fisher NI Statistical analysis of circular data. 1993 Cambridge University Press

[B28] Mardia KV Jupp PE Directional statistics. 2000 Wiley

[B29] Neggers SF Raemaekers MA Lampmann EE Postma A Ramsey NF Cortical and subcortical contributions to saccade latency in the human brain. Eur J Neurosci 2005 21 2853 2863 10.1111/j.1460-9568.2005.04129.x 15926933

[B30] Reuter B Kaufmann C Bender J Pinkpank T Kathmann N Distinct Neural Correlates for Volitional Generation and Inhibition of Saccades. J Cogn Neurosci 2009 19366286 10.1162/jocn.2009.21235

[B31] Sestieri C Pizzella V Cianflone F Romani GL Corbetta M Sequential activation of human oculomotor centres during planing of visually-guided eye movement: a combined fMRI-MEG study. Frontiers in Human Neuroscience 2008 1 1 8 10.3389/neuro.09.001.2007 PMC2525985 18958215

[B32] Derakhshan I How do the eyes move together? New understandings help explain eye deviations in patients with stroke. CMAJ 2005 172 171 173 15655233 10.1503/cmaj.1040322 PMC543974

[B33] Leigh RJ Zee DS The Neurology of Eye Movements. 1999 Oxford University Press 215 217

[B34] Priori A Bertolasi L Rothwell JC Day BL Marsden CD Some saccadic eye movements can be delayed by transcranial magnetic stimulation of the cerebral cortex in man. Brain 1993 116 Pt 2 355 367 10.1093/brain/116.2.355 8461970

[B35] Maruyama M Fenwick PBC Ioannides AA Interocular yoking in human saccades examined by mutual information analysis. Nonlinear Biomedical Physics 2010 4 Suppl 1 S10 10.1186/1753-4631-4-S1-S10 PMC2880796 20522260

